# Development of the Standardized Navigation Of Winter Mobility & Accessibility Network (SNOWMAN) course

**DOI:** 10.3389/fresc.2024.1330507

**Published:** 2024-05-09

**Authors:** Jacquie Ripat, Ed Giesbrecht, Jaimie Borisoff, Ernesto Morales, Kara-Lyn Harrison

**Affiliations:** ^1^ Department of Occupational Therapy, College of Rehabilitation Sciences, University of Manitoba, Winnipeg, MB, Canada; ^2^ Rehabilitation Engineering and Design Laboratory, MAKE+ Applied Research, British Columbia Institute of Technology, Burnaby, BC, Canada; ^3^ School of Rehabilitation Sciences, Faculty of Medicine, Université Laval, Quebec City, QC, Canada; ^4^ Applied Health Sciences, University of Manitoba, Winnipeg, MB, Canada

**Keywords:** cold climate, winter, wheelchair, rehabilitation, co-design

## Abstract

**Introduction:**

Manual wheelchairs (MWC) users have limited mobility during winter months as they encounter many environmental barriers that restrict their community participation. This paper outlines the creation and standardization of an outdoor environment designed to simulate the real-life conditions and obstacles experienced by MWC users in winter.

**Methods and results:**

This study consisted of four phases. In Phase 1, researchers used a qualitative ethnographic approach to document the specific challenges and adaptive strategies used by MWC users in winter conditions. In Phase 2, key informants with expertise in MWC winter mobility were invited to co-design the Standardized Navigation Of Winter Mobility & Accessibility Network (SNOWMAN) course. Participants reviewed draft design solutions and offered their input and suggestions to expand upon the initial design. A second co-design workshop included additional key informants, including service providers, policymakers, and professionals with expertise in landscape architecture and engineering, to validate the design solution. The workshops resulted in a detailed illustration of the SNOWMAN course, including five sections: platforms with side slopes, a miniature ice rink, curbs and curb cuts, a path with uneven winter surfaces, and modular ramps at various slopes. Phases 3 and 4 marked the conclusion of the study and involved fabrication of the SNOWMAN course and establishment of a standardized protocol for course setup and maintenance.

**Discussion:**

The project aimed to offer several additional potential benefits, supported by the various stakeholders across the study phases, that extend beyond creation of a controlled and safe environment for wheelchair users to develop their winter mobility skills. Practicing wheelchair skills in this area may assist wheelchair users in gaining confidence which may ultimately translate to increased participation in the community.

## 1 Introduction

Individuals who use manual wheelchairs (MWC) as their primary means of mobility typically experience seasonal-related accessibility barriers in the winter months (1). Environmental barriers such as icy or snow-covered surfaces and snow windrows have been reported as frequent barriers to mobility in studies of MWC users in Manitoba (2, 3). During the winter season, individuals often decrease their outdoor activities and community engagement; however, MWC users face unique obstacles that are not experienced by individuals without disabilities, which further impede their ability to go out during the winter months (4, 5). These seasonal barriers can influence both the frequency and quality of community participation. Findings from a 12-month longitudinal study of 11 wheelchair users confirmed they made fewer trips per day and wheeled shorter distances at slower speeds during non-summer (vs. summer) months, on winter days with (vs. without) snow accumulation, and on winter days when temperatures were below (vs. above) 0**°**C (1). A focus group with eight Manitobans who use wheelchairs concluded that winter community participation should be considered a right for all citizens (6). Taken together, this body of research provides evidence that inclement winter-related barriers have detrimental effects on community participation among individuals who use an MWC.

According to available data, there is an estimated 288,800 individuals in Canada who use a wheelchair or scooter for community mobility. Among these users, nearly 70% have a MWC, while approximately 15% own a power wheelchair (7). Given the high prevalence of use and more adverse impact of weather conditions and terrain, the effects of winter on MWC users is particularly relevant for investigation. Observations and recounting by MWC users have identified a variety of factors that contribute to the challenges of using a MWC in winter conditions. Ice or hard-packed snow on travel surfaces causes traction loss and slippage for both the larger rear drive wheels and the smaller front casters (1, 6, 8, 9). This loss of traction can impede forward movement or create an undesired direction change due to asynchronous wheel rotation (1). The presence of cross slopes (e.g., wheeling on a sidewalk or across a driveway) can cause the MWC to slide sideways due to gravity and reduced traction (10). These conditions require additional energy expenditure and increase the risk of collisions and tipping sideways (10). Deeper snow or snow windrows cause the small front casters to sink into the travel surface, preventing forward movement. Pushing to overcome this resistance risks tipping the MWC backward, especially when ascending or descending ramps, as maneuvering a wheelchair with raised casters (e.g., in a wheelie) makes it more susceptible to tipping (6, 8, 9). Gravity creates additional resistance when travelling on inclines and generates undesirable momentum when travelling down a slippery slope (10). Snow or slush accumulation at the lower end of the ramp (when ascending) or at the ground/ramp transition (when descending) impedes caster roll, causing sudden stop and tipping hazards (10). Unaddressed snow accumulation on level ground creates ruts that become vertical barriers to an MWC user who wants to change course (10). The user must traverse the outer margins of the ruts, which is particularly difficult with small casters, and may slide back into the rut or tip over backwards when attempting to elevate casters up and out of the rut margins (10). In summary, winter conditions reduce MWC control due to slippage, increased effort due to rolling resistance, and pose stability and safety issues.

Recommendations to enhance winter mobility for wheelchair users have focused on technology improvement (3, 5), and a few studies have delved into effective strategies and devices. Recent studies have attempted to make improvements in MWC users' experience by collating information on wheelchair mobility strategies (11) and developing protocols to make area more accessible for MWC users (12). Some research has examined experienced users navigating snow-covered ramps (8) and inexperienced users using various caster types on snow and inclines (9). However, there is a significant gap in knowledge regarding winter mobility. A scoping review by the authors found only 23 studies on winter mobility involving various mobility devices (13). A few studies of winter mobility have been conducted in simulated or controlled experimental environments (14), which lack the real-world challenges of genuine winter conditions. While useful in advancing this area of research, these settings lack the ecological validity and dynamic weather and environmental challenges of authentic winter conditions.

Currently, there is an absence of ecologically valid areas for wheelchair users to develop skills to overcome challenges faced in the winter. To address this research gap, we undertook the development of a Standardized Navigation Of Winter Mobility & Accessibility Network (SNOWMAN) course. This outdoor, winter-specific environment would incorporate a comprehensive set of conditions and obstacles/barriers commonly encountered. The intended application of the SNOWMAN course would be the development and evaluation of new devices and techniques as well as clinical interventions such as assessment and training with new and experienced mobility device users to navigate winter challenges in a safe and semi-protected, but ecologically valid, context. The purpose of this project was to conceptualize, create, and standardize use of SNOWMAN course.

## 2 Study design and team

This study employed four phases. Phase 1 documented and compiled specific real-world challenges MWC users encounter in the community and the adaptive strategies they employ for navigating winter conditions, using qualitative and observational data obtained via a go-along interview approach. Phase 2 used a Codesign Framework with key informants to identify and prioritize which real-world winter conditions were essential to the SNOWMAN course and then integrated them into a prototype design. In Phase 3, a prototype SNOWMAN course was fabricated. Phase 4 developed a study protocol, including the measurement of ambient weather conditions to standardize administration in subsequent studies, setup of course components, and measurement of component attributes. These phases are addressed sequentially below. The interdisciplinary research team consisted of researchers with backgrounds in occupational therapy, architecture, rehabilitation engineering, and wheelchair design and use.

## 3 Methods and findings

### 3.1 Phase 1: MWC user experiences with winter conditions

To identify the specific challenges and explore strategies that MWC users employ to negotiate winter conditions in the community, a qualitative ethnographic or “firsthand” research method was used (15). Ethnographic methods allow researchers to act as participant-observers (15). Using a “go-along” methodology (16) researchers were able to observe and verbally engage with MWC users to capture winter wheeling experiences in an authentic, natural setting.

Four community-dwelling individuals with spinal cord injury (SCI) participated. All participants were 18 years of age or older, had used an MWC for at least two years, and were in stable health (e.g., no known cardiac conditions). Participants were recruited through an outpatient SCI clinic and a provincial SCI advocacy organization.

Each participant's MWC was instrumented with a user point-of-view GoPro^TM^ video capture device and accompanied by a research assistant. and were asked to wheel their MWC through their own immediate community for up to 30 min. Two research assistants accompanied each participant: one as a spotter to ensure safety and the other for data collection (i.e., operating a second video camera for contextual perspective and audio recording of their experiences with the winter challenges and obstacles). In situ, participants were asked to point out challenges or obstacles they encountered and then demonstrate any strategies used to address these challenges. Immediately following the go-along activity, participants were taken to an indoor environment to engage in a 60-min audio-recorded debrief session with the research assistants. The go-along video footage was shared in an episodic fashion, stopping at specific events, and asking the participant to provide a narrative of what had occurred by highlighting and describing the challenges and strategies used to navigate the community.

The audio and video recordings from the go-along and debrief activities were not transcribed verbatim but viewed and listened to several times by the researchers to facilitate data analysis. Using NVivo 10.0 qualitative analysis software to manage and link the video and audio recorded data, the challenges and strategies identified in the video recordings and later discussed in the audio recordings were coded and categorized by the investigators. Integrative analyses of participants’ data resulted in the development of a table of challenge types and conditions. Data analysis proceeded in an inductive manner, with each subsequent participant's data entered into the table as it was collected. The validity and comprehensiveness of the final compilation of documented barriers and strategies were confirmed by a research team member not involved in data collection, who is an experienced MWC user. Barriers and challenges identified included ascending and descending snow and ice-covered slopes and curb cuts; traversing shallow, deep and slushy snow; navigating ruts and windrows; and traversing irregular snow-packed surfaces. This list and the video footage (for illustration purposes) were fed forward into Phase 2 to inform the co-design sessions.

### 3.2 Phase 2: co-design of the SNOWMAN course

To identify which specific components/obstacles were essential to include in the course, key informants with expertise in MWC winter mobility were invited to attend one of two half-day co-design sessions. Co-design refers to the act of collective creativity shared by two or more people, where the MWC user is an “expert in [their] own experience” (17). Co-design has been successfully used as a means to enhance understanding of how winter conditions affect wheelchair mobility in order to develop appropriate design solutions (18). As a guiding principle that everyone has a high creative potential and ideas on how to improve their situation, we sought diverse points of view regarding a common experience. We used an established four-step co-design framework (19), addressing the first three steps (conception of the design solution; validation of the design solution, and development of the course configuration*)* during two half-day workshops with key informants. The fourth step (testing of the course components) was addressed in Phase 3 of this study.

For *conception of the design solution,* key informants included a group of five wheelchair users and three caregivers. Each wheelchair user was at least 18 years of age, had used a wheelchair for at least two years, and had experience going out into the community with their wheelchair during winter months. Key informants were first presented with a sketched draft of possible design solutions previously developed by the research team through collaborative discussions, existing literature, and Phase 1 results. The draft design was configured for an existing sheltered outdoor space at the proposed site (a rehabilitation hospital). The draft course sketches consisted of modular sidewalk ramps (some of which were horizontal, sloped, or canted sideways) which could be integrated to create winter mobility obstacles. Each ramp consisted of a different winter condition through which wheelchair users would navigate (i.e., ice, water, deep snow, ruts, and slush).

The research team explained the project intent, and participants were invited to write, draw, or design changes on print versions of the draft design. Next, participants verbally described their ideas, with the co-design lead illustrating these in real-time. This was particularly important because once all participants understood the drawings, a common language was established, allowing everyone to contribute. The research team contributed input to concretize, give form, and translate the ideas into various design proposals. All proposed ideas were discussed collectively until a consensus proposal was achieved. By the end of the session, participants had comprehensively identified winter barriers faced by wheelchair users (see Table 1 for a list and description of barriers) and discussed how the initial draft design might be further enhanced (See Table 2 for a list of discussion topics and descriptions.).

**Table 1:** Identified barriers from wheelchair users’ and caregivers’ perspectives.

Identified barriers	Description
Ice ruts	An ice rut is a groove worn into a path from foot traffic, cars and bicycles. These ruts result in slippery and uneven surfaces, which cause difficulties when mobilizing a wheelchair.
Deep snow	Thick layers of snow require wheelchair users to exert significantly more effort when mobilizing and are often impassible
Slippery ramps	Ramps covered in ice or slush result in decreased traction while mobilizing. This creates a barrier as wheelchair users slide down ramps and require more effort or assistance to clear the obstacles.
Big chucks of snow	Chunks of hard-packed snow can make their way onto sidewalks after snow removal. These chunks impede mobilization as users attempt to clear the obstacle.
Uncleared sidewalks	After heavy snowfalls, users identified that sidewalks were not cleared. This results in users resorting to mobilizing on the roads, which makes them vulnerable to motor vehicle accidents.
Windrows	Snowplows and foot/vehicle traffic cause an accumulation of snow to pile at the end of sidewalks and curbs. This creates an obstacle that wheelchair users must pass to cross a street.
Snowy ruts and uneven paths	Tire tracks from snowplows on the sidewalk result in slippery and uneven surfaces.
Temperature-related technical problems	Cold weather may cause components of an individual's wheelchair to freeze or malfunction (e.g., wheelchair levers and clasps becoming frozen due to frigid temperatures).
Extreme temperatures	Participants identified that cold weather resulted in chances in spasticity, which caused difficulties when mobilizing.
Physical demands on caregivers	Caregivers discussed the physical demands experienced as they helped wheelchair users in the winter. Physical demands include pushing users through deep snow or ruts.
Lack of education re: winter mobility issues	Participants stated that they perceived that the general public lacked education in the difficulties wheelchair users face and how they could provide assistance.

**Table 2:** Topics that emerged in the first co-design session.

Topic	Description
Ensuring ecological validity	SNOWMAN course components should accurately represent barriers faced by wheelchair users in the winter. These included: a variety of winter surfaces (e.g., ice, slush, hard-packed snow, and deep snow); sidewalks and ramps of variable grades and angles to emulate uneven surface conditions; sidewalks with windrows (i.e., simulating those created by snowplows, car tires and footsteps); and adding curb cuts at the ends of ramps with ruts, divots and accumulations of snow, to mimic unmaintained and/or uncleared sidewalks.
Balancing safety and variability	The course should balance safety with the inclusion of realistic yet difficult obstacles. The range of obstacles should enable people to progressively build their skills comfortably and access varying levels of challenge for skill development.
Making it fun	While the main purpose of the SNOWMAN course is training and device development, participants suggested adding areas where users could enjoy winter leisure activities, such as a miniature ice rink for adapted hockey or curling.

**Figure 1:**
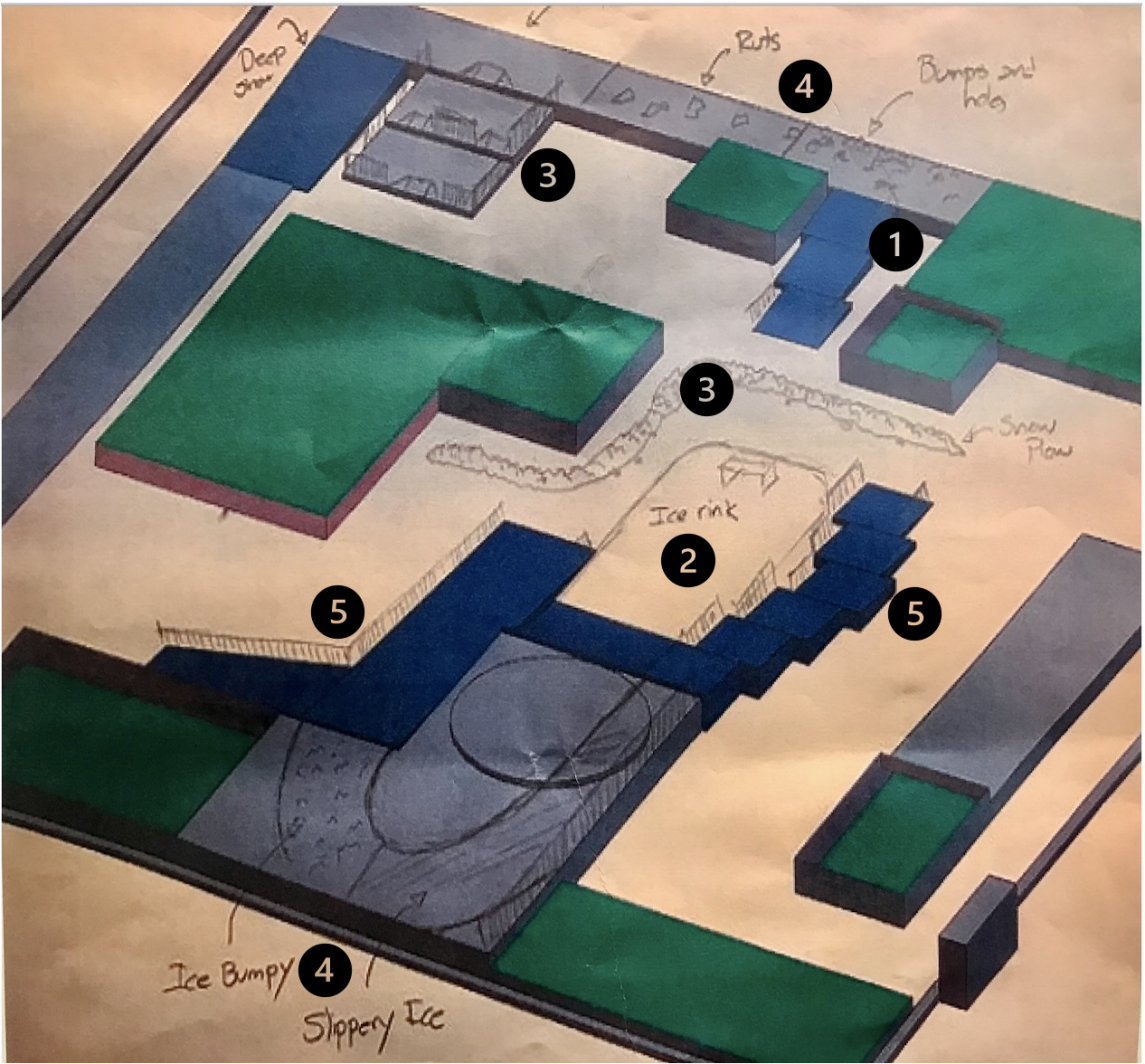
Initial consensus design proposal. Numbers align with section descriptions provided in manuscript text.

The consensus proposal was consolidated by the co-design lead into a more refined graphic depiction (See Figure 1 for a detailed illustration of the results). The course included five sections: (1) platforms with side slopes similar to what wheelchair users encounter propelling on the sidewalk) and covered with a variety of conditions, including hard-packed snow, ice and slush; (2) a miniature ice rink where users can practice mobilizing on icy surfaces; (3) curbs and curb cuts that each feature a different winter condition as well as windrows piled at the bottom of the entrance to mimic unmaintained/uncleared sidewalks; (4) a path of uneven winter surfaces that wheelchair users typically encounter in the community such as ice ruts, deep snow, and large chunks of snow, and (5) modular ramps at various slopes with different winter conditions.

For *validation of the design solution and development of course configuration,* a second co-design workshop was conducted with six additional key informants (i.e., service providers, policymakers, and professionals with expertise in landscape architecture and engineering). The refined consensus design from session one was presented for criticism, enrichment, and confirmation, with the goal of enhancing the design and setting parameters that would result in a realistic, practical, and feasible solution (See Table 3 for details of discussion topics). They expressed enthusiasm for the concept and identified additional uses for such a course but also proposed some feasibility concerns regarding setup, maintenance, and safety. For instance, they advocated reducing the proposed grade of slopes to be within accessibility guidelines rather than the steep “ski slope” that had been proposed by the first group. They identified the need to add rails to higher platforms to prevent accidental falls and the need to create barriers to areas that might be hazardous if individuals were able to use the course without support or supervision. Based on the proposed conceptual design in session one and subsequent support and refinement provided in session two, we felt confident in our stakeholders' validation of the SNOWMAN course. The validated results from the second co-design session were defined using Solidworks 3D design software (Dassault Systems, Waltham, MA), producing specifications for the construction of the course components (not shown).

**Table 3:** Topics that emerged during the second co-design session.

Topic	Description
Exposure and education	Clinicians endorsed the importance of introducing clients to winter conditions and challenges, for instance: exposing clients to cold weather to assess its impact on spasticity; navigating ramps that are manageable in summer but challenging in winter; encountering sand and salt used for preventing slips on roads and sidewalks and how it effects wheelchair components; and dealing with snow glare. The winter obstacle course would allow exposure to winter-related barriers encountered by wheelchair users, fostering educational opportunities and skill development in a semi-controlled setting.
Expanding beyond wheelchair use	Participants saw additional benefits of the winter obstacle course in providing opportunities for clients with gait aids to practice mobilizing in the winter, providing caregivers with training/education, trialling new equipment, and training/educating city employees.
Feasibility	Participants generated important questions to ensure that the winter obstacle course would be realistic, such as: • Who will maintain the course? • How will wheelchair users dry their equipment following trials? • What safety precautions will be in place when using the area? • What are the rules for using the area? • What types of equipment and training do clinicians require before taking clients into the area?

### 3.3 Phase 3: fabrication of the SNOWMAN course

**Figure 2:**
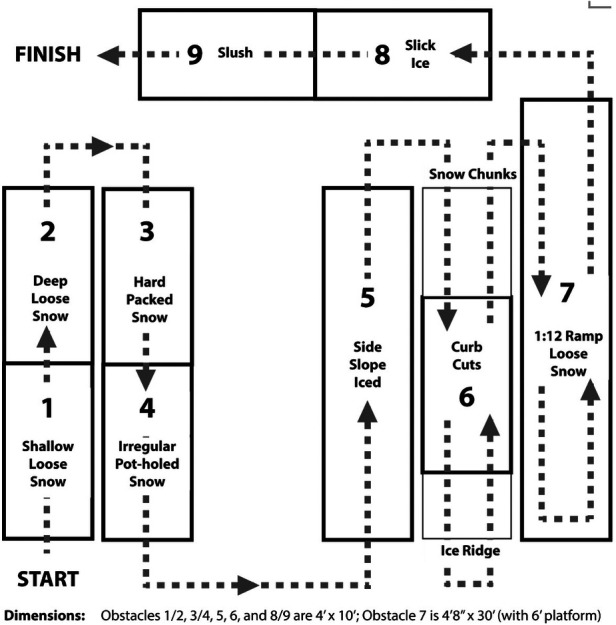
Schematic of the SNOWMAN course.

**Figure 3:**
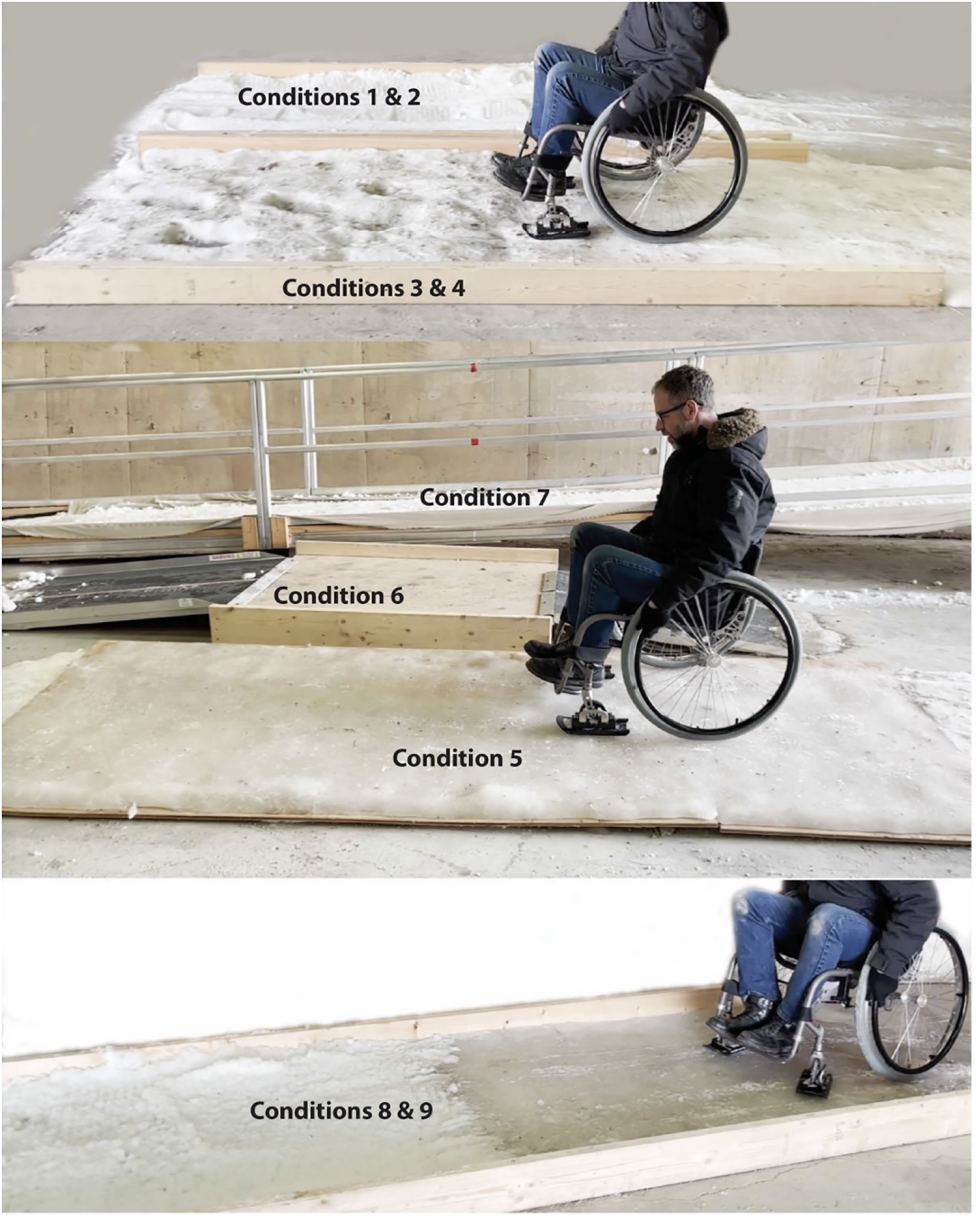
Photo of SNOWMAN course in January 2023.

Due to cost, space, and resource constraints, a reduced-scope prototype of the SNOWMAN course was fabricated in winter 2022–2023. We constructed the standardized course in a large uninsulated covered shed which was unheated and fully exposed on one wall. The course was created in Winnipeg, Manitoba, Canada, where the average winter temperature between November–March is −10.7°C and the average snowfall is 20.9 mm. The course included a defined pathway, taking wheelchair users through a series of nine challenging winter conditions: (1) shallow (2”) loose snow, (2) deep (4”) loose snow, (3) hard-packed snow, (4) irregular pot-holed snow, (5) iced 5° side slope/sidewalk, (6) two curb cuts with snow chunks and an ice ridge at the two approaches, (7) a 1 : 12 (5°) snow-covered ramp (1/2” snow) with snow accumulation at the base, (8) a slick icy flat surface, and (9) a slushy surface. See Figure 2 for a schematic of the course and Figure 3 for a series of photos of the course in January 2023.

### 3.4 Phase 4: development of a protocol for standardization of course setup and measurement of component attributes and ambient weather conditions

As there was no literature available regarding the creation of winter conditions, our research team undertook a process of fabricating snow and ice compositions required for each course component. This process was necessarily iterative to achieve conditions that would mimic real-world winter obstacles. Once these were satisfactory, we created a protocol manual to ensure consistent replication. Because the course is situated outdoors, we were unable to control for ambient temperature and humidity; however, most conditions of the course were under investigator control and made as consistent as possible for future testing, training, or evaluation sessions. For instance, water was sprinkled on the icy surfaces with a watering can prior to testing to ensure a comparable coefficient of friction. Loose snow was added to the shallow and deep snow obstacles to ensure correct depth when this had been reduced due to sublimation and were subsequently groomed using a bow rake. Slushy snow was prepared by mixing windshield washer fluid (containing anti-freeze) and soft snow in specific proportions to ensure homogenous and consistent hardness and density. New snow was added to the ramp and groomed for each trial as snow left in place tended to get hard and freeze to the ramp. Additionally, the snow chucks were replaced at the bottom of the curb cuts whenever these were disturbed during use.

Given the specified obstacle composition and the course location outdoors, we also developed a standardized protocol for testing the controllable obstacle snow conditions as well as the uncontrollable weather conditions so these could be documented in a consistent way. Using valid and reliable methods identified in the literature (19), we developed a standardized protocol (available upon request from the first author) for measuring the constructed obstacle snow conditions as well as the ambient weather conditions. This protocol was completed by a research assistant (RA), who was hired to set up, maintain, and groom the course components and to document conditions in preparation for subsequent data collection. The standardized protocol involved measuring and recording ambient temperature and humidity; snow depth, surface temperature, hardness, and density on snow-covered obstacles; and coefficient of friction on icy surfaces.

## 4 Discussion

Based on expert stakeholders' lived experiences with winter conditions, we successfully created a standardized outdoor wheelchair mobility course consisting of nine unique winter obstacles and a course construction and maintenance protocol. Following the co-design sessions, it was clear that wheelchair users, caregivers, clinicians, professionals, and policymakers were supportive of project SNOWMAN.

Through the project, we identified the need to develop a space that incorporated multiple and varied winter conditions routinely encountered to enable comprehensive comparison and evaluation that could differentiate between different mobility devices and solutions. Furthermore, we identified the need to create a context that was of sufficient space to replicate real-life challenges (i.e., enough of the obstacle to capture authentic encounters and to replicate the authenticity of overground propulsion). Although standardization of conditions in such an outdoor course may not mirror precisely reproducible indoor laboratory environments, a strength of this winter test course is its applicability to varying, real-world conditions that are not captured in an ecologically valid way by traditional research settings.

The development of the SNOWMAN course provides an opportunity to address the evaluation of wheelchair mobility skills in diverse contexts. Existing outcome measures, such as the Wheelchair Skills Test (19, 20) are comprehensive in scope of mobility skills included but may not address the same breadth of environments and surfaces, particularly in standardized testing. SNOWMAN may provide the opportunity to use such outcome measures in a complementary way or potential adaptation of existing measures for evaluation in winter environments.

The project aims to offer several additional potential benefits, supported by the various stakeholders across the study phases, that extend beyond creation of a controlled and safe environment for wheelchair users to develop their winter mobility skills. Practicing wheelchair skills in this area may assist wheelchair users in gaining confidence which may ultimately translate to increased participation in the community. SNOWMAN can serve as a testing ground for mobility device manufacturers to innovate and improve winter-ready devices. Clinicians can use this space to prepare clients for winter challenges, offering practical techniques and education. Additionally, it allows clinicians to observe clients using new mobility equipment and gather evidence to support equipment funding. Lastly, it could provide a training platform for civic employees responsible for snow clearing and accessible transportation, potentially improving overall winter accessibility in the community.

## 5 Conclusion

The development of SNOWMAN represents a step toward addressing a critical research gap in providing areas for wheelchair users to develop skills and overcome challenges faced during winter. Through this process, we have created a unique environment that provides a context for winter-specific mobility device innovation and testing. This project has been a collaborative effort involving researchers, wheelchair users, caregivers, clinicians, professionals, and policymakers, all of whom have demonstrated strong support for this initiative. The next step in this research is to use the SNOWMAN course to evaluate whether it is an ecologically valid course that can distinguish between various wheelchair configurations and user abilities. Overall, SNOWMAN is a groundbreaking initiative with the potential to positively impact the lives of wheelchair users and the broader community by enhancing winter mobility and accessibility.

## Data Availability

The raw data supporting the conclusions of this article will be made available by the authors, without undue reservation.
